# Long-term mortality and cause of death in people with tuberculosis compared with matched controls with influenza or non-typhoid salmonellosis in Australia: a retrospective cohort study

**DOI:** 10.1136/bmjph-2024-001848

**Published:** 2026-03-02

**Authors:** Anthony Byrne, Kenneth Ng, Ellen Donnan, Jennifer Pett, Neil Heron, Javier Huarat, Serena Koenig, Ben J Marais

**Affiliations:** 1 Thoracic Medicine, St Vincent’s Hospital Sydney, Darlinghurst, New South Wales, Australia; 2 St Vincent’s Clinical School, University of New South Wales Medicine & Health, Sydney, New South Wales, Australia; 3 Department of Medicine, University of New South Wales Medicine & Health, Sydney, New South Wales, Australia; 4 Tuberculosis Program, NSW Health, St Leonards, New South Wales, Australia; 5 Royal Melbourne Hospital, BioGrid Australia Ltd, Parkville, Victoria, Australia; 6 Brigham and Women’s Hospital, Harvard Medical School, Boston, Massachusetts, USA; 7 Marie Bashir Institute for Infectious Diseases and Biosecurity, University of Sydney SDN, Sydney, New South Wales, Australia

**Keywords:** Public Health, Secondary Prevention, Tuberculosis, Comorbidity

## Abstract

**Introduction:**

Tuberculosis (TB) patients are thought to experience excess long-term morbidity and mortality. However, the long-term mortality of people with TB has not been compared with other common infections to assess their overall and disease-specific causes of death.

**Methods:**

All people diagnosed with TB between 2000 and 2015 in New South Wales, Australia, were matched by age, sex and year of infection to notified cases of influenza or non-typhoid salmonellosis. Patients were linked to the Australian mortality register to assess date and cause of death, up until the end of 2022. The comparative mortality ratio (CMR) for each cohort was calculated and Kaplan-Meier survival curves constructed. The standardised mortality rate (SMR) was estimated using national mortality data.

**Results:**

The CMR of 7386 people with past TB was marginally increased (1.16 with 95% CI 1.10 to 1.22) compared with non-typhoid salmonellosis but reduced compared with influenza (0.88 with 95% CI 0.84 to 0.93). Most deaths occurred within 10 years of the notified infection. For TB patients, 1162 of 1331 people (87.3%) died in the 10 years after TB diagnosis. Risk factors for long-term mortality among TB patients included age over 40 years, male sex, sputum smear-positive and intracranial disease. Respiratory causes of death, including chronic obstructive pulmonary disease, lung cancer and pneumonia, were increased for TB patients, while influenza patients suffered more cardiovascular deaths. The SMR for TB patients (compared with the Australian general population) was increased by 18.7 times for infectious causes of death, 2.8 times for respiratory causes and 1.4 times for cancer-related mortality.

**Conclusions:**

Despite successful treatment, people with TB often experience post-TB sequelae and increased long-term mortality, with increased risk of respiratory causes of death. This highlights the need for appropriate post-TB respiratory follow-up, particularly in the first 10 years after TB diagnosis.

**WHAT IS ALREADY KNOWN ON THIS TOPIC**

- Despite successful treatment, tuberculosis survivors in both low-middle and high-income settings appear to have increased long-term mortality compared to national mortality rates.

**WHAT THIS STUDY ADDS**

- This study (uniquely) identifies the most common causes of death for three distinct infectious diseases (two respiratory and one gastrointestinal). We demonstrate increased long-term mortality (for people with notified tuberculosis and influenza compared to non-typhoid salmonellosis). For people with tuberculosis, death from respiratory-specific diseases including infection, chronic obstructive pulmonary disease (COPD) and lung cancer was significantly higher than those with prior influenza or non-typhoid salmonella.

**HOW THIS STUDY MIGHT AFFECT RESEARCH, PRACTICE OR POLICY**

- Screening for respiratory comorbid disease such as COPD among people with a history of tuberculosis may provide opportunities for interventions to reduce respiratory-specific long-term mortality.

## Introduction

 People with tuberculosis (TB) disease are known to have an increased mortality despite successful antimicrobial treatment.^1^ However, it is unclear what specific medical conditions cause death, or how the long-term mortality of TB patients compares with other notifiable infections. In 2024, an estimated 10.7 million children and adults suffered TB disease globally, with approximately 155 million people experiencing post-TB sequelae equating to 58 million disability-adjusted life-years.^2 3^ Mortality during the TB treatment period is uncommon in low TB incidence settings like Australia. However, as most TB programmes discharge patients after treatment completion, deaths that occur in the months or years following ‘cure’ are not recorded and common causes of death among TB survivors are unknown.

TB survivors have an increased risk of permanent chronic lung disease, including chronic obstructive pulmonary disease (COPD) and bronchiectasis.^4^ While effective antimicrobial TB treatment saves many lives and improves lung health, the long-term sequelae, including post-TB mortality, are rarely monitored or reported.^5 6^ Few studies have assessed long-term post-TB mortality, particularly in settings where an accurate cause of death can be determined.^7^ Increased long-term mortality among TB survivors when compared with death rates in the general population has been shown, but not compared with other common respiratory and non-respiratory infections that are also notifiable.^1 7^ Moreover, the specific causes and timing of death among TB survivors remain unclear.

The COVID-19 pandemic increased awareness of excess short-term mortality from respiratory viral infections, but long-term mortality is unknown. Limited evidence from hospitalised patients with other respiratory infections (community-acquired pneumonia) suggests an increased cardiovascular risk.^8 9^ The systemic pro-inflammatory response and ‘vascular cascade’ following SARS-CoV-2 virus can lead to post-acute COVID-19 sequelae that can last for months to years.^10^ While long-term mortality following COVID-19 is not yet available, influenza is a notifiable disease in Australia and the availability of rapid PCR tests within hospital and community settings provides an opportunity to study long-term health effects following influenza infection compared with TB disease. To date, there are no studies that have assessed the effect of influenza infection on long-term mortality. Because influenza and TB primarily affect the respiratory system and are known to increase the risk of COPD, pulmonary hypertension and other associated respiratory disease, we also included a non-respiratory notifiable infection (non-typhoid salmonellosis) as a comparator group.^11^ While no single infectious disease comparator truly represents a ‘healthy control’, the two diseases chosen are both common globally and generally considered to cause acute illness. All three infectious diseases use the same well-established data collection system for notifiable diseases in New South Wales (NSW), Australia. In NSW, the Public Health Act (2010) mandates notification directly from diagnostic laboratories. For TB, routine programme data collected includes treatment initiation details, adherence and end of treatment outcome, but there is no current mechanism to collect post-TB health outcome data.

In this study, we compared the long-term mortality of TB patients with matched influenza and non-typhoid salmonellosis cases and explored the most common causes of death in each group. To better understand the timing of when post-TB deaths occur, and what risk factors are associated with long-term mortality following TB diagnosis, we asked (1) ‘What is the long-term mortality of TB patients compared with those diagnosed with influenza or non-typhoid salmonellosis and when do these deaths occur?’ and (2) ‘Are particular causes of death over-represented in any disease group?

## Materials and methods

A retrospective, population-based cohort study of all TB cases diagnosed in NSW, Australia, from 1 January 2000 to 31 December 2015 was performed. The start date coincided with the initiation of the Notifiable Conditions Information Management System (NCIMS) in NSW and the end date was chosen to provide a minimum 5-year ‘survival window’. Influenza PCR testing was widely available at this time, although influenza was not notifiable until 2001. The Australian Institute of Health and Wellbeing (AIHW) manages the National Death Index (NDI), which collates information on deaths from jurisdictional birth, death and marriages registers including cause of death based on the International Classification of Diseases (ICD) version 10.

All patient episodes of TB, influenza and non-typhoid salmonellosis (adults and children) between 1 January 2000 and 31 December 2015 were extracted from the (NSW) NCIMS database on 15 July 2021. Patient episodes of influenza and non-typhoid salmonellosis were excluded from the potential control pool if that person had been diagnosed with TB at any time during the study period. Each TB episode, (henceforth called patient) was matched by age (within 5 years), sex and year of notification (same year). All patients were then linked to the AIHW NDI to obtain date and cause of death. The 20-year average death rate for the TB cohort was calculated and compared with the 2022 specific cause of death rates for the Australian general population provided by AIHW (henceforth named Standardised Mortality Rate (SMR)).

All people with a diagnosis of TB were included in the study population, from the time of their diagnosis. WHO defined treatment outcomes for each TB patient were not included in the analysis. However, historically, the NSW TB programme achieved treatment success (‘treatment complete’ or ‘cure’) in over 90% of people diagnosed with TB. Death during TB treatment is very rare (<1% of treated cases) and mostly occurs in older people (≥65 years of age). To determine and compare all-cause mortality experienced by different disease groups, mortality ratios were calculated by dividing the observed number of deaths (such as for TB cases) by the observed number of deaths in one of the two control groups. The 1-year, 2-year, 5-year and 10-year survival rates were calculated and displayed on a Kaplan-Meier curve, stratified by relevant study groups. A log-rank test was performed to assess differences in long-term survival.

For secondary study outcomes, the causes of death were categorised into the following categories—neoplastic, cardiovascular, infection-related, respiratory and other. Cause-specific comparative mortality ratios (CMRs) were calculated for these five groups. Among TB patients who died, the timing of the deaths was classified as on-treatment, post-treatment and post-mortem (where the diagnosis of TB occurred after the time of death). HRs were calculated via a Cox regression to determine which variables were associated with long-term survival and to what extent. SAS V.9 for windows was used for data analysis.

## Results

We identified 7386 patient episodes of TB diagnosed during the study period, matched with 6386 influenza patients and 7353 non-typhoid salmonellosis patients. Baseline characteristics of each group are presented in table 1. All were evenly split between males and females, with an age range of 0–100 years and median age of 38 for both the TB and influenza cohorts and 37 for the non-typhoid salmonellosis cohort. Patient numbers were similar with the exception of influenza patients during the first time period (2000–2005) as influenza only became notifiable as a public health condition in 2000.

**Table 1:** Baseline characteristics of tuberculosis patients, and matched influenza and non-typhoid salmonella cohorts

Characteristic	Tuberculosis patients	Reference cohorts	
		Influenza	Non-typhoid salmonellosis
Total number (N)	7386	6386	7353
Year of disease, n (%)			
2000–2005	2566 (34.7)	1684 (26.4)	2534 (34.5)
2006–2010	2449 (33.2)	2331 (36.5)	2449 (33.3)
2011–2015	2371 (32.1)	2371 (37.1)	2370 (32.2)
Males, n (%)	3912 (53.0)	3399 (53.2)	3890 (52.9)
Total observation time (years)	85 207	67 055	87 671
Mean observation time in years (SD)	11.5 (5.6)	10.5 (4.9)	11.9 (5.3)
Median age at diagnosis, years (IQR)	38 (27–58)	38 (27–59)	37 (26–57)
Age-group at diagnosis, n (%)			
0–9	157 (2.1)	159 (2.5)	176 (2.4)
10–19	330 (4.5)	455 (7.1)	415 (7.1)
20–29	1964 (26.6)	1436 (22.5)	1938 (26.4)
30–39	1425 (19.3)	1278 (20.0)	1391 (18.9)
40–49	956 (12.9)	778 (12.2)	935 (12.7)
50–59	818 (11.1)	748 (11.7)	814 (11.1)
60–69	627 (8.5)	593 (9.3)	672 (9.1)
70–79	631 (8.5)	528 (8.3)	603 (8.2)
80–89	407 (5.5)	365 (5.7)	356 (4.8)
≥90	71 (1.0)	46 (0.7)	53 (0.7)

Table 2 summarises the sociodemographic and clinical characteristics of TB patients. There were 12.4% Australian born, 26.4% permanent residents, 8.0% either international students or refugees, and 46.6% with no visa status recorded (missing). Most TB was laboratory confirmed (77.0%) with predominantly pulmonary disease (60.1%). Of pulmonary TB with sputum smear status available, 29.5% (1307/3330) had smear-positive TB. Almost all TB patients (94.7%) were newly diagnosed and 92.5% (4710/5094) were fully susceptible.

**Table 2:** Sociodemographic and clinical characteristics of tuberculosis patients (N=7386)

Characteristic	n (%)
Site of disease	
Pulmonary (including pulmonary with other sites) Sputum smear positive (N=4438) Sputum smear negative (N=4438)	4438 (60.1) 1307 (29.5) 3131 (70.5)
Extra pulmonary	2944 (39.8)
Unknown	4 (0.1)
TB disease classification	
New active	7041 (95.0)
Recurrence	332 (4.5)
Unknown	40 (0.5)
Diagnosis and laboratory findings	
Microbiological confirmation Fully sensitive to first-line TB medications Isoniazid mono-resistant Rifampicin mono-resistant MDR XDR TB Other resistance pattern	5094 (69.0) 4710 (92.5) 250 (4.9) 18 (0.4) 54 (1.1) 3 (0.1) 59 (1.2)
HIV infection status	
Positive	123 (1.7)
Negative	3248 (44.0)
Not tested or testing declined	2301 (31.1)
Unknown	1714 (23.2)
Migrant status	
Australian born	916 (12.4)
Permanent resident	1951 (26.4)
Refugee/humanitarian visa	49 (0.7)
Overseas student or visitor visa	698 (9.5)
Other or unknown	3772 (51.1)

MDR, multidrug-resistant; XDR TB, extensively drug-resistant tuberculosis.

**Figure 1:**
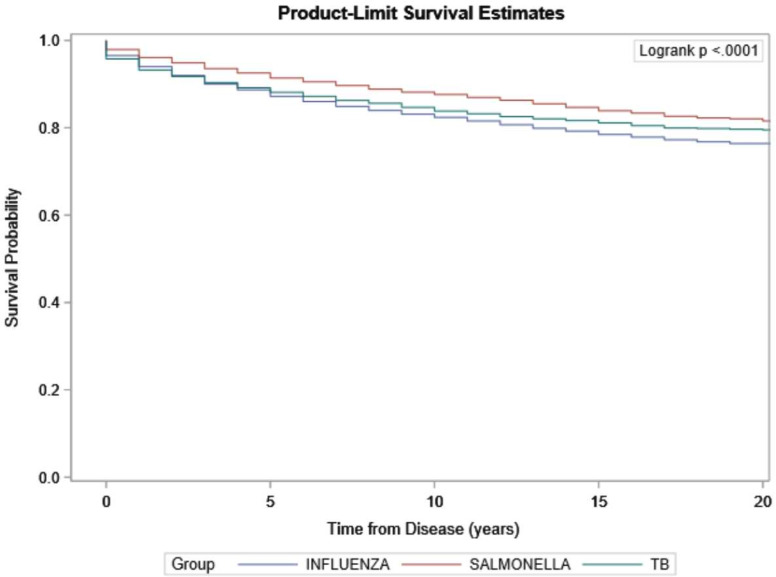
Kaplan-Meier survival curve of tuberculosis patients compared with matched influenza and non-typhoid salmonellosis cohorts. *p<0.001 using a log-rank test stratified by the three study groups of Influenza, non-typhoid salmonellosis and *Mycobacterium tuberculosis*. TB, tuberculosis.

Kaplan-Meier curves (figure 1) illustrate the survival of TB patients compared with people with influenza and salmonella from the time of diagnosis. These demonstrate significantly reduced survival rates for influenza and TB patients compared with matched non-typhoid salmonella (log-rank test p<0.05). The 1-year, 2-year, 5-year and 10-year survival rates of TB patients were 93.2%, 91.8%, 88.2% and 83.8%, respectively. The log-rank test found a significant difference between the survival curves for the TB and salmonella cohorts. When only the cases with a negative survival time were excluded, the crude 1-year, 2-year, 5-year and 10-year survival rate of TB cases from notification date was 93.5%, 92.1%, 88.7% and 84.1%, respectively.

The overall number of deaths was slightly more in the TB cohort (1331 people, 18.0%), compared with the influenza cohort (1304 people, 20.4%) with fewer deaths among the Salmonella cohort (1145 people, 15.6%). The CMR of 7386 people with TB was increased (1.16 with 95% CI 1.10 to 1.22) compared with those with non-typhoid salmonella infection, but reduced compared with influenza (0.88 with 95% CI 0.84 to 0.93). Online supplemental figure S1 illustrates the timing of death following diagnosis, demonstrating that most deaths occur within 10 years of the initial infection for all three cohorts. Among 7386 TB patients there were 1331 (18.0%) deaths; 1160 (87.2%) occurred post-TB treatment and 169 (12.8%) while on TB treatment. In comparison, 1145 (15.6%) non-typhoid salmonellosis patients (age, gender, year of infection matched), and 1304 influenza patients (20.4%) died during this study period. There were significantly more TB patients than non-typhoid salmonellosis patients that died during the 5–10-year period (CMR 1.59 with 95% CI 1.45 to 1.72). There was no difference in the time-specific CMR for TB and influenza patients during this time (CMR 0.99 with 95% CI 0.90 to 1.07).

Table 3 provides HRs for variables associated with all-cause mortality in TB patients. After adjusting for variables in the multivariate cox-regression model, being male, older than 59 years old, having an unknown HIV status, positive sputum smear result, intracranial disease or an unknown disease classification was associated with poorer prognosis (greater mortality). Deaths among people with extrapulmonary TB were reduced compared with those with pulmonary TB. Drug resistance was not found to be associated with increased risk of mortality in this study after adjustment for covariates, although numbers were small.

**Table 3:** Unadjusted and adjusted HRs of all-cause mortality risk in tuberculosis patients

Characteristic	HR (95% CI)	P value	Adjusted HR (95% CI)	P value
Sex				
Female	1		1	
Male	1.82 (1.63 to 2.04)	<0.0001	1.52 (1.35 to 1.70)	<0.0001
Age at diagnosis				
<20 years	1		1	
20–39 years	0.73 (0.36 to 1.49)	0.3868	0.96 (0.47 to 1.97)	0.9127
40–59 years	6.56 (3.37 to 12.79)	<0.0001	7.27 (3.72 to 14.20)	<0.0001
≥60 years	52.86 (27.42 to 101.9)	<0.0001	50.91 (26.37 to 98.29)	<0.0001
HIV infection status				
Positive	0.64 (0.33 to 1.24)	0.1825	0.69 (0.36 to 1.36)	0.2917
Sputum smear				
AFB negative	1		1	
AFB positive	1.25 (1.08 to 1.45)	0.0032	1.19 (1.02 to 1.39)	0.0252
Intracranial disease				
No	1		1	
Yes	1.47 (1.08 to 2.00)	0.0135	2.07 (1.50 to 2.86)	<0.0001
Drug resistance				
Fully drug sensitive	1		1	
M/XDR	0.31 (0.12 to 0.84)	0.0204	0.43 (0.16 to 1.14)	0.0907
Other resistance profiles	0.72 (0.54 to 0.95)	0.0226	0.81 (0.61 to 1.08)	0.1439
Site of TB disease				
Pulmonary	1		1	
Extra pulmonary	0.52 (0.46 to 0.58)	<0.0001	0.71 (0.61 to 0.82)	<0.0001
Pulmonary plus other sites	0.89 (0.74 to 1.08)	0.2541	1.00 (0.82 to 1.21)	0.9778
TB disease classification				
First time TB	1		1	
Recurrent TB	1.56 (1.26 to 1.95)	<0.0001	1.10 (0.88 to 1.37)	0.3886

AFB, acid fast bacilli; MDR, multidrug resistant; TB, tuberculosis; XDR, extensively drug resistant.

Table 4 reflects the recorded ICD-10 codes for causes of death from the five disease categories. Neoplastic death was not significantly increased among the TB cohort compared with non-typhoid salmonellosis or influenza (CMR 1.04; 95% CI 0.93 to 1.15 and CMR 1.04; 95% CI 0.93 to 1.15, respectively). However, there were significantly more lung cancer deaths (respiratory and intrathoracic organs), with 87/324 (26.9%). Of 87 deaths from lung cancer, most (68, 78.2%) occurred within 5 years of TB diagnosis.

**Table 4:** Cause-specific mortality rate in tuberculosis patients compared with matched non-typhoid salmonella and influenza cohorts

Causes of death	Time since infection diagnosis	ICD-10	TB cohort deaths	Salmonella cohort CMR (95% CI)*	Influenza cohort CMR (95% CI)*
Neoplastic	–	C00–D49	324	1.04 (0.93 to 1.15)	1.04 (0.93 to 1.15)
Respiratory and intra-thoracic organs	–	C30–39	87	1.60 (1.27 to 1.94)	1.12 (0.89 to 1.36)
	<2 years	–	38	1.58 (1.08 to 2.08)	0.82 (0.56 to 1.08)
	2–5 years	–	30	1.57 (1.01 to 2.13)	2.00 (1.28 to 2.71)
	5–10 years	–	11	1.83 (0.75 to 2.9)	1.36 (0.56 to 2.16)
	>10 years	–	8	1.59 (0.49 to 2.7)	0.99 (0.3 to 1.67)
Mesothelioma and soft tissue	–	C45–49	7	1.00 (0.26 to 1.73)	0.76 (0.2 to 1.32)
Urinary tract	–	C64–68	18	1.38 (0.74 to 2.02)	2.22 (1.2 to 3.25)
Lymphoid and haematological	–	C81–96	50	1.00 (0.72 to 1.27)	0.96 (0.69 to 1.23)
Cardiovascular		I00–I99	243	0.76 (0.67 to 0.86)	0.68 (0.59 to 0.77)
Ischaemic heart disease	–	I20–I25	106	0.79 (0.64 to 0.94)	0.61 (0.49 to 0.72)
Pulmonary circulation	–	I26–28	6	1.19 (0.24 to 2.15)	0.58 (0.12 to 1.04)
Cerebrovascular disease	–	I60–69	72	0.98 (0.76 to 1.21)	1.00 (0.77 to 1.24)
Infection-related	–	A00–B99, J00–J22	226	5.62 (4.89 to 6.36)	1.97 (1.72 to 2.23)
Sequelae of TB	–	B90	10	–	2.88 (1.10 to 4.67)
Influenza and pneumonia	–	J09–18	32	1.99 (1.3 to 2.68)	0.56 (0.37 to 0.76)
Respiratory	–	J30–99	173	1.91 (1.63 to 2.2)	0.64 (0.55 to 0.74)
Emphysema	–	J43	4	–	0.31 (0.01 to 0.62)
Other COPD	–	J44	87	1.80 (1.43 to 2.18)	0.55 (0.43 to 0.66)
Asthma	–	J45	6	1.49 (0.3 to 2.69)	0.65 (0.13 to 1.17)
Other	–	All other codes	365	0.94 (0.84 to 1.03)	0.80 (0.72 to 0.88)

* CMR compared with the reference group of the tuberculosis cohort.

CMR, comparison mortality rate; COPD, chronic obstructive pulmonary disease; ICD-10, International Classification of Diseases version 10; TB, tuberculosis.

Cardiovascular death (I00–I99) was common overall and more frequent among people with previously notified influenza. Infection-related deaths (A00–B99, J00–J22) were more likely among TB patients compared with the salmonella and influenza groups (CMR 5.62; 95% CI 4.89 to 6.36 and 1.97; 95% CI 1.72 to 2.23 respectively). Of the 226 infection-related deaths reported in the TB cohort, 32 were due to influenza pneumonia. COPD deaths were the predominant respiratory cause of death among TB patients (87/173, 50.3%), with 66.7% (58/87) occurring within 5 years of TB diagnosis. Overall, 173 non-infectious respiratory causes of death (including COPD, asthma and lung cancer) were significantly increased among the TB cohort compared with non-typhoid salmonellosis (CMR 1.91; 95% CI 1.63 to 2.2), but not compared with influenza.

Online supplemental figure S2 displays cause-specific death outcomes for TB patients compared with the general Australian population death rate (over 20 years, per 100 000). The TB cohort demonstrated a death rate 18.7 times higher than the general population from infectious causes (ICD A00–B99, not age-matched). For respiratory-specific causes of death such as COPD (JOO–J99), the death rate for TB patients was 2.8 times higher than the general population.

## Discussion

With total numbers of global TB increasing^1^ and TB survivors facing a significant risk of death despite microbiological cure,^1 7 12^ long-term TB-related mortality is highly relevant.^13^ Our large Australian study with a very long follow-up of up to 20 years and comprehensive specific cause of death notification data found significantly more long-term mortality among both TB and influenza patients compared with matched patients with non-typhoid salmonellosis. Relative to deaths in the Australian general population, these deaths among former TB patients, particularly due to infectious and respiratory-specific causes, appear quite high. We found the SMR for TB patients in NSW to die from an infectious cause to be almost 19 times that for the Australian general population and almost three times for respiratory-specific causes.

Excess deaths among people with TB have been reported in high and low TB incidence settings.^6 7 12^ However, socioeconomic factors are important confounders given TB is more prevalent among poor and marginalised populations.^6^ The SMR observed in Vietnam was 3.33 compared with 1.86 in Denmark, for example.^7 12^ As Australia is a high-income country with socioeconomic protections, lower GINI index and low rates of cigarette smoking, this may explain the relatively low CMR of 1.16 (not SMR) seen in our study. Compulsory screening for active TB among migrants and no-cost TB care in Australia is likely to result in earlier diagnosis of less severe disease.^14^ Previous studies showed delayed TB diagnosis increases risk of COPD and mortality, with more intensive care admissions if (TB) treatment was delayed by >1 month.^15 16^ Earlier TB diagnosis in our cohort is expected because two thirds had negative (acid fast bacilli) sputum microscopy (with positive culture), correlating with earlier disease and a less-severe phenotype.^17^ Another important reason for the lower overall mortality estimate in our study is the suspected loss of long-term mortality data from those TB patients that were not Australian citizens or permanent residents, who may have returned to their country of origin during the 20-year follow-up period. Their deaths would not be captured in the national mortality registry and our results would bias to the null, giving a more conservative estimate of long term TB mortality.

Almost all TB deaths occurred following TB treatment completion, at a time when most TB programmes have discharged patients without further follow-up. People with TB (median age 38 years at diagnosis) experienced increased death due to a respiratory cause, and over 50% was death due to COPD, which supports other studies demonstrating a strong link between TB and chronic respiratory disease.^4 11^ This indicates a role for more active post-TB disease lung function monitoring. Recent data from a subgroup of the same NSW cohort showed significantly increased respiratory symptoms and abnormal lung function among TB survivors despite successful treatment.^18^ While cigarette smokers and people with COPD are at increased risk of developing TB, accumulating evidence suggests TB itself contributes to the global COPD burden, particularly among non-smokers.^19^,^21^ Post-TB lung disease is now clearly defined with the newly published research definition, requiring an antecedent diagnosis of pulmonary or pleural disease with two or more of the following: persistent respiratory symptoms, abnormal lung function and abnormal chest imaging with typical features of radiological post-TB disease.^22^

The second International Post TB Symposium called for greater awareness, research and advocacy for TB survivors who suffer impaired lung health and greater mortality.^21 23^ With new TB treatment regimens shortening the duration of antimicrobial therapy, there is an urgent need to follow up TB patients beyond treatment completion to ensure a durable microbiological cure.^24^ Without lung function and other non-microbiological outcomes in clinical trials, it is impossible to compare the merits of new TB treatment regimens as they pertain to lung health.^24^ With more infectious death post-TB, vaccination for diseases such as influenza, COVID-19 and pneumococcus for TB patients would be logical and consistent with international clinical standards.^25^

HIV and drug resistance (including multi-drug resistant (MDR)-TB) were not significant risk factors for long-term mortality in our study, unlike other studies from high TB and HIV incidence settings.^7 15 16^ However, people with HIV-TB coinfection and MDR-TB in our study were few (both below 2%). Universal healthcare and routine whole genomic sequencing is available in Australia, facilitating individualised TB treatment regimens and high rates of microbiological cure (>90%).^26^ This likely explains our negative findings for these risk factors, which differ from a Chinese setting that reported an adjusted HR of 3.50 for MDR-TB.^27^ Other studies found diabetes was associated with unfavourable treatment outcomes^28^ and increased frequency of cavitary lung lesions.^29^ This information was not captured in our database, and we were thus unable to account for it in our analysis.

Importantly (and surprisingly), cancer was the predominant cause of death among TB patients in our study, with one quarter of neoplastic-related deaths due to lung cancer. While cancer deaths were not increased overall, compared with the comparison cohorts, TB patients were much more likely to die from lung cancer in the 2–5 years after TB diagnosis. Previous research noted an association between TB and lung cancer but has not shown when lung cancer occurs in relation to TB diagnosis.^30^ With low dose CT scanning now available to screen for lung cancer in high-risk smokers across North America and Europe,^31^ the identification of pulmonary nodules or masses in previously treated TB patients is sure to become a more common problem. Consideration could be given to a baseline CT scan in TB patients at TB treatment completion to identify suspicious pulmonary nodules and may also diagnose bronchiectasis and emphysema. Older patients (>65 years) and those with a smoking history would benefit most from a CT scan as they also have higher mortality risk.

Our study is the first to compare long-term mortality of TB patients to other infections, and the first to demonstrate excess long-term mortality post viral influenza. With millions contracting influenza each year, this finding is important and suggests greater attention to postacute care of influenza patients. In Australia, there were over 225 000 cases of influenza in 2022, with 0.1% mortality from the acute illness,^32^ but there is almost no research on postacute sequelae of influenza.^33^ Limited data suggests more cardiovascular events in the months after acute influenza.^9^ One study found an increased short-term mortality among elderly nursing home residents;^34^ another from Mexico showed greater respiratory and cardiovascular mortality among children under 5 and adults over the age of 60 years.^35^ Increased long-term risk of cardiovascular death postinfluenza from our study indicates the need for further research. In the meantime, attention to vascular risk factors for post-influenza patients seems prudent.

Strengths of our study include its population-based design and large cohort of over 7000 TB patients in NSW, Australia. Matching by age, sex and year of notification to two comparison groups of infections reduced confounding. Few studies have assessed age-matched and sex-matched reference groups, and none in this way.^1 7 12^ second, as a high-income country, Australia has a robust system of recording deaths that require medical certification.^36^ Although not as accurate as formal autopsy, this method is considered reliable and is not subject to recall bias like ‘verbal autopsy’, used in other studies.^7^ Third, the follow-up period of up to 20 years is matched by few studies.^12^ Lastly, the method of calculating the CMR allowed for variation in mortality over time by matching with year of infection, age and gender. Prior studies calculated the SMR by using their respective national mortality rates as ‘control’,^37 38^ but these vary significantly between low, middle and high-income countries and do not accommodate mortality rate changes over time or optimal matching between cohorts.

A major study limitation is the fact that we were unable to consider socioeconomic variables in the analysis. TB disease risk is strongly correlated with socioeconomic status, which also has a strong influence on lung disease and mortality risk. Despite being constrained by the information available and unable to consider socioeconomic variables, our analysis demonstrated excess mortality among people with a history of TB compared with those with non-typhoid salmonellosis. Australia is a high-income country with a low GINI coefficient (reflecting limited inequality) that would reduce the impact of socioeconomic disparity.^39^ However, national level equality may not correlate to individual level disadvantage, thus socioeconomic hardship and relevant lifetime exposures remain important risk factors to consider and their absence limits the inferences that can be made from our analysis.

To improve the rigour of our mortality comparisons, we also compared death rates in the TB cohort to the Australian general population. With a median age of 38 years at TB diagnosis, the TB study cohort was slightly younger than the Australian general population (median age 38.5 years in 2022).^40^ It is notable that the death rate among our TB cohort was greater than the Australian general population, despite the slight age mismatch introducing bias in the opposite direction. The effect of COVID-19 is another factor that might have biased 2022 mortality data, since we compared post-TB deaths over a 20 year period to those in the Australian general population in 2022. However, this would have biased towards increased mortality in the 2022 general population cohort, strengthening the findings of increased SMR in the TB cohort.

Another major study limitation is the fact that the majority (61%) of TB patients were neither Australian citizens nor permanent residents, indicating an increased likelihood of missing mortality data. While some TB survivors would have stayed in Australia, many may have left. Death occurring outside of Australia in someone who is not an Australian citizen or permanent resident would not be captured in the Australian NDI. Residency and visa status were not recorded for the other two disease cohorts, but it is expected that the vast majority would have been Australian citizens or permanent residents. This data collection gap may explain why our TB mortality rate was less than in other studies.^7 12 41^ Similar to previous limitations, this would have biased against increased mortality in the TB cohort, which gives us confidence in the direction of the observed trends even if effect size quantification may be compromised.

We conclude that people diagnosed with TB in Australia experience increased long-term mortality compared with the general public and matched patients diagnosed with non-typhoid salmonellosis. Over the 20-year observation period, most post-TB deaths occurred within 10 years of TB diagnosis. People with TB were more likely to die from respiratory infections, COPD and lung cancer, which suggests that better post-TB lung health monitoring and better respiratory infection prevention through vaccination require consideration. Lung health monitoring may include lung function testing at TB treatment completion and chest CT scans of high-risk patients, but more research is needed to know which interventions will have the greatest value in particular subgroups. The observation that influenza patients experienced greater long-term death and cardiovascular disease also warrants further research.

## Supplementary material

10.1136/bmjph-2024-001848 online supplemental file 1

10.1136/bmjph-2024-001848 online supplemental file 2

## References

[1] Romanowski K, Baumann B, Basham CA (2019). Long-term all-cause mortality in people treated for tuberculosis: a systematic review and meta-analysis. Lancet Infect Dis.

[2] World Health Organization (2025). Global tuberculosis report. https://www.who.int/teams/global-programme-on-tuberculosis-and-lung-health/tb-reports/global-tuberculosis-report-2025.

[3] Dodd PJ, Yuen CM, Jayasooriya SM (2021). Quantifying the global number of tuberculosis survivors: a modelling study. Lancet Infect Dis.

[4] Byrne AL, Marais BJ, Mitnick CD (2015). Tuberculosis and chronic respiratory disease: a systematic review. Int J Infect Dis.

[5] Marais BJ, Chakaya J, Swaminathan S (2020). Tackling long-term morbidity and mortality after successful tuberculosis treatment. Lancet Infect Dis.

[6] Raviglione M, Marais B, Floyd K (2012). Scaling up interventions to achieve global tuberculosis control: progress and new developments. Lancet.

[7] Fox GJ, Nguyen VN, Dinh NS (2019). Post-treatment Mortality Among Patients With Tuberculosis: A Prospective Cohort Study of 10 964 Patients in Vietnam. Clin Infect Dis.

[8] Stotts C, Corrales-Medina VF, Rayner KJ (2023). Pneumonia-Induced Inflammation, Resolution and Cardiovascular Disease: Causes, Consequences and Clinical Opportunities. Circ Res.

[9] Nguyen JL, Yang W, Ito K (2016). Seasonal Influenza Infections and Cardiovascular Disease Mortality. JAMA Cardiol.

[10] Phetsouphanh C, Jacka B, Ballouz S (2024). Improvement of immune dysregulation in individuals with long COVID at 24-months following SARS-CoV-2 infection. *Nat Commun*.

[11] Byrne A, Al-Hindawi Y, Plit M (2025). The prevalence and pattern of post tuberculosis lung disease including pulmonary hypertension from an Australian TB service; a single-centre, retrospective cohort study. BMC Pulm Med.

[12] Christensen A-SH, Roed C, Andersen PH (2014). Long-term mortality in patients with pulmonary and extrapulmonary tuberculosis: a Danish nationwide cohort study. Clin Epidemiol.

[13] Allwood BW, van der Zalm MM, Amaral AFS (2020). Post-tuberculosis lung health: perspectives from the First International Symposium. Int J Tuberc Lung Dis.

[14] New south wales department of health annual tuberculosis reporting. https://www.health.nsw.gov.au/Infectious/tuberculosis/reports/2024/Pages/default.aspx%20https://www.health.nsw.gov.au/Infectious/tuberculosis/reports/2023/Pages/default.aspx.

[15] Lee C-H, Lee M-C, Lin H-H (2012). Pulmonary tuberculosis and delay in anti-tuberculous treatment are important risk factors for chronic obstructive pulmonary disease. PLoS One.

[16] Zahar JR, Azoulay E, Klement E (2001). Delayed treatment contributes to mortality in ICU patients with severe active pulmonary tuberculosis and acute respiratory failure. Intensive Care Med.

[17] Nguyen M-VH, Levy NS, Ahuja SD (2019). Factors Associated With Sputum Culture-Negative vs Culture-Positive Diagnosis of Pulmonary Tuberculosis. JAMA Netw Open.

[18] Byrne A, Al-Hindawi Y, Rigava S (2025). Persistent respiratory symptoms and increased reactance among successfully treated australian tb survivors compared to healthy controls.

[19] Caballero A, Torres-Duque CA, Jaramillo C (2008). Prevalence of COPD in five Colombian cities situated at low, medium, and high altitude (PREPOCOL study). Chest.

[20] Amaral AFS, Coton S, Kato B (2015). Tuberculosis associates with both airflow obstruction and low lung function: BOLD results. Eur Respir J.

[21] Nightingale R, Carlin F, Meghji J (2023). Post-TB health and wellbeing. Int J Tuberc Lung Dis.

[22] Bisson GP, Allwood B, Byrne A (2025). Post-tuberculosis lung disease: a case definition for use in research studies. Lancet Infect Dis.

[23] Allwood BW, Nightingale R, Agbota G (2024). Perspectives from the 2^nd^ International Post-Tuberculosis Symposium: mobilising advocacy and research for improved outcomes. IJTLD Open.

[24] Byrne A, Allwood B, Schoeman I (2023). Post tuberculosis”: the urgent need for inclusion of lung health outcomes in tuberculosis treatment trials. Eur Respir J.

[25] Migliori GB, Marx FM, Ambrosino N (2021). Clinical standards for the assessment, management and rehabilitation of post-TB lung disease. Int J Tuberc Lung Dis.

[26] NSW TB Program (2019). Tuberculosis in new south wales: surveillance report 2018. https://www.health.nsw.gov.au/Infectious/tuberculosis/Publications/2018-tb-report.pdf.

[27] Liu Y, Zheng Y, Chen J (2018). Tuberculosis-associated mortality and its risk factors in a district of Shanghai, China: a retrospective cohort study. int j tuberc lung dis.

[28] Alisjahbana B, Sahiratmadja E, Nelwan EJ (2007). The effect of type 2 diabetes mellitus on the presentation and treatment response of pulmonary tuberculosis. Clin Infect Dis.

[29] Shaikh MA, Singla R, Khan NB (2003). Does diabetes alter the radiological presentation of pulmonary tuberculosis. Saudi Med J.

[30] Hwang SY, Kim JY, Lee HS (2022). Pulmonary Tuberculosis and Risk of Lung Cancer: A Systematic Review and Meta-Analysis. J Clin Med.

[31] Baldwin DR, O’Dowd EL, Tietzova I (2023). Developing a pan-European technical standard for a comprehensive high-quality lung cancer computed tomography screening programme: an ERS technical standard. Eur Respir J.

[32] Australian Government Department of Health and Aged Care (2022). National 2022 influenza season summary. https://www.health.gov.au/sites/default/files/2022-12/aisr-2022-national-influenza-season-summary.pdf.

[33] Troeger CE, Blacker BF, Khalil IA (2019). Mortality, morbidity, and hospitalisations due to influenza lower respiratory tract infections, 2017: an analysis for the Global Burden of Disease Study 2017. Lancet Respir Med.

[34] Gaillat J, Chidiac C, Fagnani F (2009). Morbidity and mortality associated with influenza exposure in long-term care facilities for dependent elderly people. Eur J Clin Microbiol Infect Dis.

[35] Salto-Quintana JN, Rivera-Alfaro G, Sánchez-Ramos EL (2019). Post-pandemic influenza-associated mortality in Mexico. Pathog Glob Health.

[36] Australian institute of health and welfare (2012). National death index (NDI), data quality statement.

[37] Blöndal K, Rahu K, Altraja A (2013). Overall and cause-specific mortality among patients with tuberculosis and multidrug-resistant tuberculosis. Int J Tuberc Lung Dis.

[38] Ranzani OT, Rodrigues LC, Bombarda S (2020). Long-term survival and cause-specific mortality of patients newly diagnosed with tuberculosis in São Paulo state, Brazil, 2010-15: a population-based, longitudinal study. Lancet Infect Dis.

[39] Australian institute of health and welfare website. https://www.aihw.gov.au/reports/australias-welfare/international-comparisons-of-welfare-data.

[40] Australian bureau of statistics website. https://www.abs.gov.austatisticspeoplepopulation#:~:text=Australia’s%20population%20in%202022%20(26,between%2043.8%20and%2047.6%20years.

[41] Wang X-H, Ma A-G, Han X-X (2015). Survival and associated mortality risk factors among post-treatment pulmonary tubercolosis patients in the northwest of China. Eur Rev Med Pharmacol Sci.

