# Supplementary material for: Long-term mortality and cause of death in people with tuberculosis compared with matched controls with influenza or non-typhoid salmonellosis in Australia: a retrospective cohort study
Source: BMJ Public Health. 2026 Mar 2;4(1):e001848. doi: 10.1136/bmjph-2024-001848 (PMC12959072; doi:10.1136/bmjph-2024-001848)
Supplement: online supplemental file 1 [file bmjph-4-1-s001.pdf]

Figure S1

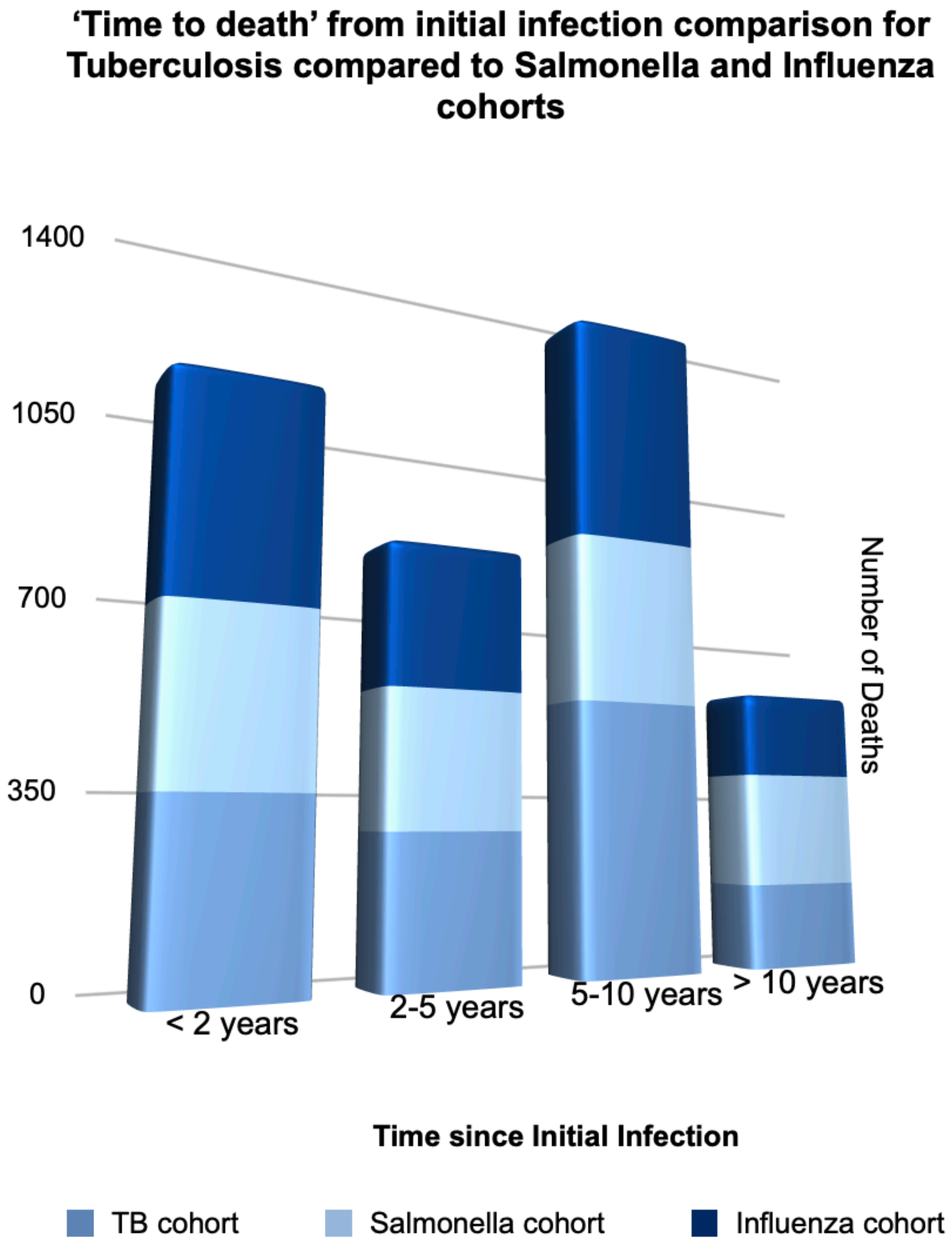

'Time to death' from initial infection comparison for Tuberculosis compared to Salmonella and Influenza cohorts
1400
1050
700
350
0
< 2 years
2-5 years
5-10 years
> 10 years
Number of Deaths
Time since Initial Infection
TB cohort
Salmonella cohort
Influenza cohort
