# Supplementary figures and images for: Long-term mortality and cause of death in people with tuberculosis compared with matched controls with influenza or non-typhoid salmonellosis in Australia: a retrospective cohort study
Source: BMJ Public Health. 2026 Mar 2;4(1):e001848. doi: 10.1136/bmjph-2024-001848 (PMC12959072; doi:10.1136/bmjph-2024-001848)

Figure S2

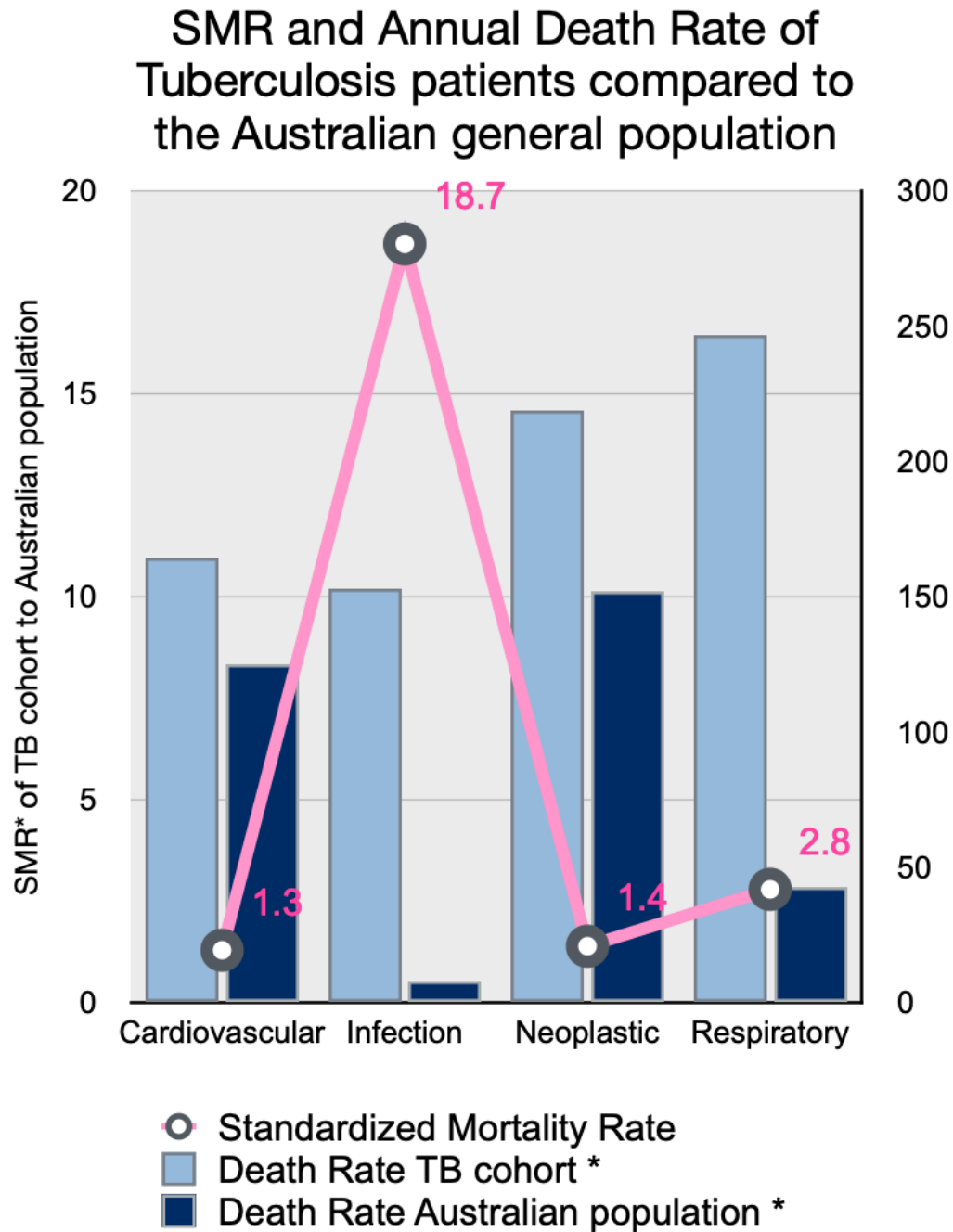

2nd Y axis \* Annual Death rate per 100,000

Supplement: online supplemental file 2 [file bmjph-4-1-s002.pdf]
